# Population genetic structure of the carrot weevil (*Listronotus oregonensis*) in North America

**DOI:** 10.1111/eva.13343

**Published:** 2022-02-01

**Authors:** Marianne Bessette, Dave T. Ste‐Croix, Jacques Brodeur, Benjamin Mimee, Annie‐Ève Gagnon

**Affiliations:** ^1^ Saint‐Jean‐sur‐Richelieu Research and Development Centre Agriculture and Agri‐Food Canada Saint‐Jean‐sur‐Richelieu QC Canada; ^2^ Département de sciences biologiques Institut de recherche en biologie végétale Université de Montréal Montreal QC Canada

**Keywords:** apiaceous host plants, carrot weevil, genetic structure, genotyping‐by‐sequencing, geographic distance, mtDNA COI

## Abstract

Population genetic studies of insect pests enhance our ability to anticipate problems in agroecosystems, such as pest outbreaks, insecticide resistance, or expansions of the host range. This study focuses on geographic distance and host plant selection as potential determinants of genetic differentiation of the carrot weevil *Listronotus oregonensis*, a major pest of several apiaceous crops in North America. To undertake genetic studies on this species, we assembled the first complete genome sequence for *L. oregonensis*. Then, we used both haplotype discrimination with mitochondrial DNA (mtDNA) and a genotyping‐by‐sequencing (GBS) approach to characterize the genetic population structure. A total of 220 individuals were sampled from 17 localities in the provinces of Québec, Ontario, Nova Scotia (Canada), and the state of Ohio (USA). Our results showed significant genetic differences between distant populations across North America, indicating that geographic distance represents an important factor of differentiation for the carrot weevil. Furthermore, the GBS analysis revealed more different clusters than COI analysis between Québec and Nova Scotia populations, suggesting a recent differentiation in the latter province. In contrast, we found no clear evidence of population structure associated with the four cultivated apiaceous plants tested (carrot, parsley, celery, and celeriac) using populations from Québec. This first characterization of the genetic structure of the carrot weevil contributes to a better understanding of the gene flow of the species and helps to adapt local pest management measures to better control this agricultural pest.

## 1 INTRODUCTION

Agronomic practices such as crop selection, tillage, or pesticide applications cause strong ecological disturbances on insect pest populations (Brust & King, 1994; Wezel et al., 2014). These conditions favor the selection of adapted individuals that may eventually lead to failure of pest management strategies (Corrêa et al., 2019; Gould, 1991). In agroecosystems, natural selection may act locally and generate different patterns of geographic variation in life history traits of herbivorous insects, including developmental rate, phenology (Renner & Zohner, 2018), host plant specificity (Jiggins & Bridle, 2004), insecticide resistance (Ffrench‐Constant et al., 2000), or dispersal capacity (Mazzi & Dorn, 2012). Knowledge of the genetic structure of insect populations can thus contribute to the design and implementation of locally, better adapted pest management strategies (Anderson et al., 2016; Rollins et al., 2006).

Genetic variations within and among populations result from a number of evolutionary drivers: mutation, gene flow, genetic drift, and selection (Coyne, 1992; Pinho & Hey, 2010). For herbivorous insects, their capacity to disperse and colonize new habitats is a major determinant leading to divergent genetic population structure (Mazzi & Dorn, 2012). Efficient dispersal capacity facilitates gene flow between regions, thereby reducing genetic structuring (Bohonak, 1999; Broquet & Petit, 2009; Kim & Sappington, 2013; Roderick, 1996). In contrast, the genetic diversity of species having poor dispersal capacity is often limited to the diversity associated with a single or a few events of colonization (bottleneck and founder effects) and further reduced following strong genetic drift. Such a pattern can result in significant population structuring (Hanks & Denno, 1994; Slatkin, 1987). Abiotic factors such as climate and landscape also affect the dispersal capacity of insects and trigger isolation (Grez & Villagran, 2000). Although geographic distance represents one of the most important factors in genetic differentiation of populations, host suitability and availability can also lead to host‐associated genetic differentiation (Angelella et al., 2019; Antwi et al., 2015; Hood et al., 2020). This evolutionary pathway constitutes a continuum of differentiation that can generate genetic changes across populations leading to reproductive isolation and sympatric speciation (Drès & Mallet, 2002; Forbes et al., 2017). The host plant can act as the main determinant of population differentiation alone (Groman & Pellmyr, 2000; Silva‐Brandão et al., 2018) or in combination with geographic isolation (Agosta, 2006). From an applied perspective, pest species having distinct genotypes associated with different host plants or cultivars may differ in their vulnerability to pest control methods (Machado et al., 2008; Martel et al., 2003; Shufran et al., 2000).

Native to North America, the carrot weevil, *Listronotus oregonensis* (LeConte) [Coleoptera; Curculionidae], is mainly distributed in the Great Lakes region (Justus & Long, 2019). This pest attacks Apiaceae, notably carrot (*Daucus carota* L. subsp. *sativus*), parsley (*Petroselinum crispum* L.), and celery (*Apium graveolens* L.) (Chandler, 1926). Distribution across its range appears to be highly fragmented and would match the regions where its host plants are cultivated. However, wild plants such as wild carrot (*D. carota* L.), wild parsnip (*Apium petroselinum* L.), water parsnip *Sium suave* Walter (Apiaceae), common plantain (*Plantago major* L.), lance‐leafed plantain (*Plantago lanceolate* L.) (Plantaginaceae), wild turnip (*Brassica rapa* L.) (Brassicaceae), and several *Rumex* species (*Rumex* spp.) (Polygonaceae) can also be exploited by the carrot weevil and may contribute to maintain local populations nearby agricultural areas (Boivin, 2013). The carrot weevil completes one to three generations per year depending on the latitude (Boivin, 1999), and climate warming tends to increase voltinism in northern regions (Boivin, 2013; Telfer et al., 2018). Adults have a low dispersal capacity, moving mainly by walking despite being winged (Wright & Decker, 1957). Females lay their eggs on petioles, and larvae typically burrow into roots. Significant crop damage can be observed, reaching up to 90% in the absence of pest control strategies (Boivin, 1999). Carrot weevil management relies mainly on adult scouting, synchronized applications of foliar insecticides, and crop rotations (Gagnon et al., 2021; Justus & Long, 2019). Levels of damage have likely increased recently across North America, suggesting a change in *L. oregonensis* phenology or local adaptation of populations (e.g., pesticide resistance) that could disrupt pest control (Telfer et al., 2018).

The main objective of this study was to investigate the genetic structure of *L. oregonensis* populations across its range in North America based on two complementary approaches: haplotype discrimination with mitochondrial DNA (mtDNA) and genotyping‐by‐sequencing (GBS). In addition, a whole‐genome assembly was produced and released as the first draft genome for this species. More specifically, these genomic resources for the carrot weevil allowed us to examine the role of geographic distance and host plant selection in structuring populations.

## 2 MATERIALS AND METHODS

### 2.1 Insect sampling

**FIGURE 1:**
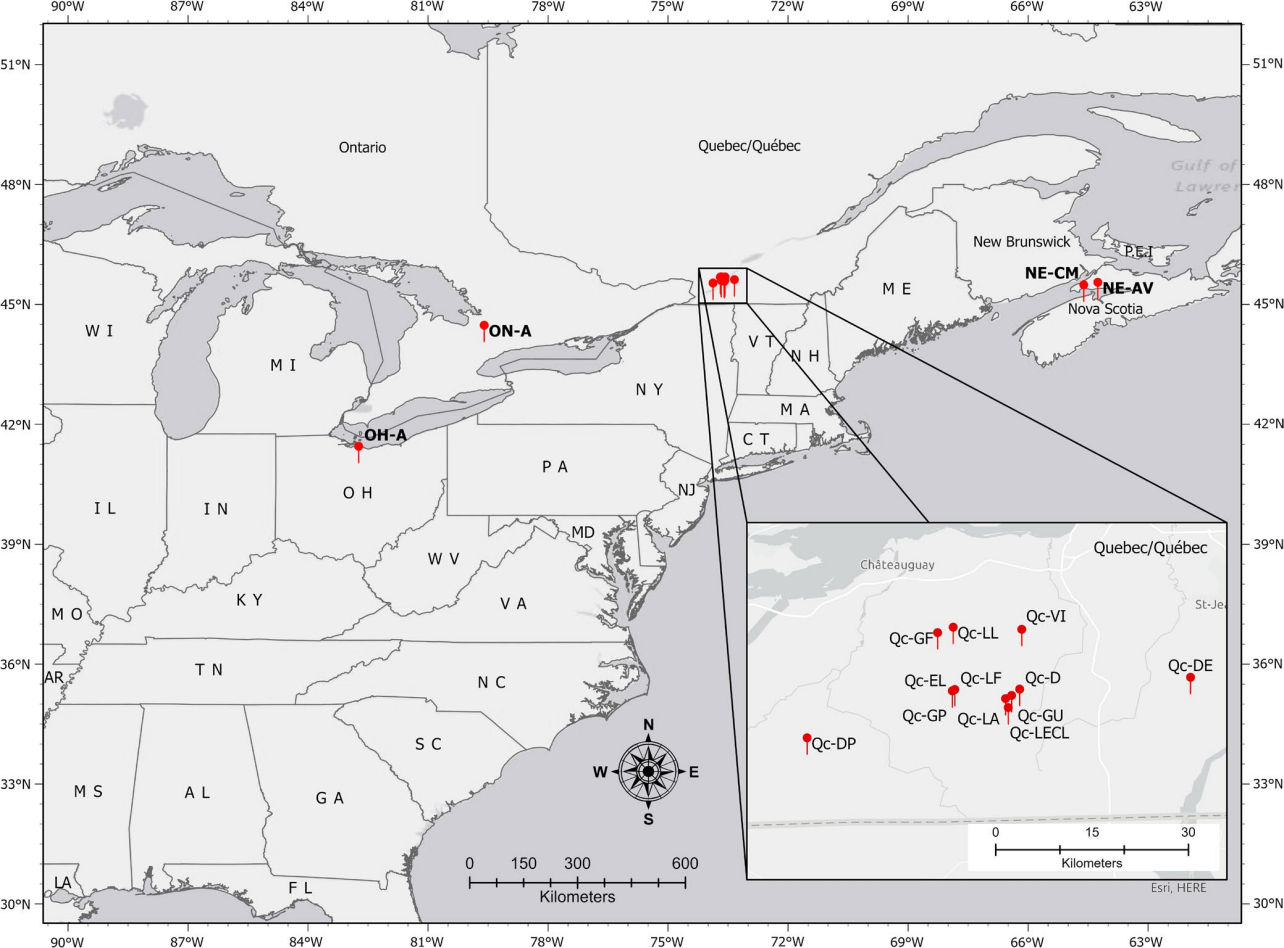
*Listronotus oregonensis* sampling locations across northeastern North America

In 2018 and 2019, carrot weevil adults were collected across eastern Canada and in Ohio, USA (Figure 1), on four crops of the apiaceous family: carrot (*D. carota* L. var. *sativus*), celery (*A. graveolens* L. var. dulce), parsley (*P. crispum* L.), and celeriac (*A. graveolens* L. var. *rapaceum*), using carrot‐bait traps (Boivin, 1985) placed at the edge of commercial fields. This sampling technique is recommended for all Apiaceae crops (Boivin, 1985; Justus & Long, 2019). Sampling sites were chosen according to (i) their geographic location across the range of the carrot weevil, (ii) their history of infestation by *L. oregonensis*, and (iii) species of host plant (Table S1). In the province of Québec, a finer geographic scale analysis was carried out focusing sampling efforts on weevils from various host plant species. We also tested individuals from a rearing colony established at the Saint‐Jean‐sur‐Richelieu Research and Development Centre of Agriculture and Agri‐Food Canada, with initial specimens collected at the experimental farm located at Sainte‐Clotilde‐de‐Chateauguay. This laboratory population is maintained on carrot roots and has never been restocked with other field‐collected individuals for 15 years. A total of 220 individuals were sampled from 17 localities distanced by a maximum of 2000 km, from Ohio to Nova Scotia (Table S1). Adult weevils were preserved in 1.5‐ml Eppendorf‐type vials with 95% ethanol until DNA extraction. Voucher specimens (one individual per locality) were also deposited at the Ouellet‐Robert Entomological Collection (Université de Montréal, Montréal, Québec, Canada) (QMOR57165–QMOR57181).

### 2.2 DNA extraction

A DNA extraction protocol was adapted from the DNeasy Blood and Tissue Kit (Qiagen). For each individual, the six legs were removed from the body and frozen at −80°C for at least one hour to facilitate tissue grinding. Legs were then crushed in extraction buffer directly into the 1.5‐ml Eppendorf tube using a pestle (Ultident Scientific). Samples were incubated overnight at 56°C (16 h) in the lysing buffer, and all subsequent steps followed the recommendations from the extraction kit. DNA was eluted in 100 μl of distilled water and stored at −20°C. Concentration and purity of extracted DNA were analyzed with a NanoDrop 2000 spectrophotometer (Thermo Fisher Scientific) and a Qubit fluorometer (Invitrogen). DNA amount per sample was normalized to between 100 and 500 ng/μl for mtDNA (COI) sequencing and to 2 ng/μl for the GBS approach.

### 2.3 Genome sequencing and assembly

Fifty weevils from the rearing colony, aged between 2.5 and 3.5 months, were used to extract gDNA using the method described above. The concentration was assessed on the Qubit fluorometer (Invitrogen) system using the Qubit dsDNA HS Kit. Prior to library preparation, extraction quality was confirmed on the Agilent 2100 Bioanalyzer using the Agilent DNA 7500 Kit. Libraries were then prepared for both long‐read and short‐read sequencing. The sequencing libraries for the Oxford Nanopore MinION were prepared using the Ligation Sequencing Kit (SQK‐LSK110) following the manufacturer’s recommendations. The resulting library was sequenced on R9.4.1 MinION flow cells (Oxford Nanopore Technologies) for 72 h or to pore exhaustion. A total of five libraries, from the same DNA extraction, were generated and sequenced. Short‐read sequencing library preparation was carried out at McGill and Genome Québec Innovation Center (Montréal, Québec, Canada) and sequenced (paired‐end 2 × 150 bp) on a single lane of NovaSeq 6000 (Illumina).

### 2.4 Bioinformatic processing

Sequences from the five nanopore runs were merged before being simultaneously basecalled, trimmed of adaptor sequences, and filtered based on quality scores using Guppy (v.3.6; Oxford Nanopore) with the flags qscore_filtering and min_qscore 7 set. The Illumina short reads were trimmed of adaptors and filtered based on a minimum Q score of 30 using Fastp (v.0.20.1; Chen et al., 2018). Quality filtered reads were assembled into draft contigs using a two‐step assembly approach. First, the long‐read assembly pipeline wtdbg2 (v2.5; Ruan & Li, 2020) was used to generate an initial set of contigs using the parameter –X 25, ‐x preset1, ‐g 1.3G, ‐L 8k, ‐p 21, ‐‐edge‐min 2, and ‐‐rescue‐low‐cov‐edges set. In a second assembly, the short reads were combined with a 25× subset of the longest nanopore reads using the hybrid assembler HASLR (v.0.8a1; Haghshenas et al., 2020) under default settings. Both assemblies were then merged into a single draft using Quickmerge (v.0.3; Chakraborty et al., 2016). The resulting draft was then scaffolded using LINKS (v 1.8.7; Warren et al., 2015) prior to polishing using TGS‐GapCloser (v1.1.1; Xu et al., 2020) and pilon (v.1.23; Walker et al., 2014), with long‐read and short‐read sequences, respectively. We then used BlobTools (v1.1.1; Laetsch & Blaxter, 2017) to remove any bacterial contamination from the final assembly before assessing completeness using BUSCO (v4.0.6; Seppey et al., 2019) with the Arthropoda lineage specified. The carrot weevil genome size was also estimated by the J. Spencer Johnston’s laboratory at the Texas A&M University using flow cytometry on six adult males and six adult females following a protocol modified from Hare and Johnston (2012).

### 2.5 Genotyping approaches

Haplotype discrimination with mtDNA COI and a GBS approach were used as two complementary methods to characterize genetic population structures of the carrot weevil. The combination of genotyping methods contributes to a more global comprehension of the genetic population structure by analyzing regions (mtDNA vs genomic DNA) having different molecular evolutionary rates (Xia et al., 2018). GBS analyses show more recent genetic changes from single‐nucleotide polymorphisms (SNPs) on nuclear DNA, while COI mtDNA can go much further back in the evolution timescale (Avise, 1991; Brumfield et al., 2003). Moreover, mtDNA, transmitted only by the mother, is not subjected to genetic recombination and is more sensitive to the founder effect (Avise, 1991; Birky et al., 1989; DeSalle & Giddings, 1986). By using these two approaches, we aimed to assess the genetic differences between populations of the carrot weevil and to estimate whether these differences occurred on a more distant or recent timescale.

### 2.6 Mitochondrial DNA (COI) sequencing

Partial sequence of the mitochondrial cytochrome c oxidase subunit I (COI) was amplified by PCR using the universal primers LCO1490 (5′‐GGT CAA CAA ATC ATA AAG ATA TTG G‐3′) and HCO2198 (5′‐TAA ACT TCA GGG TGA CCA AAA AAT CA‐3′) (Folmer et al., 1994). PCRs were performed in a 25 μl volume containing 2.5 units of *Taq* DNA polymerase, 1 × Qiagen elution Buffer (containing 1.5 mM MgCl_2_), 0.5 μl of dNTP (10 mM), 1.5 μl of 10 μM forward and reverse primers, 0.5 μl of MgCl_2_ (25 μM), and 2 μl of template DNA. PCRs were realized using a Mastercycler^®^ thermo‐cycler (Eppendorf) with the following cycler conditions: 94°C during 3 min (initial denaturation); 5 cycles at 94°C for 30 s (denaturation), 45°C for 30 s (annealing), and 72°C for 30 s (extension); followed by 35 cycles at 94°C for 30 s (denaturation), 48°C for 30 s (annealing), and 72°C for 30 s (extension); and 72°C for 10 min (final extension). PCR product sizes were visualized by electrophoresis on a 1.5% agarose gel (BioShop). The amplified PCR fragments were sequenced with a 3730xl DNA Analyzer (Applied Biosystems) at the Genome Québec Innovation Center and McGill University (Montréal, Québec, Canada). Then, forward and reverse sequences were assembled and edited to 685 bp using CLC Main Workbench V20.0 (Qiagen). Sequence alignment was performed on MEGA X, using MUSCLE with the default options (Kumar et al., 2018). A total of 206 individuals from 17 localities were analyzed.

### 2.7 Genotyping‐by‐sequencing (GBS)

Each carrot weevil individual was genotyped by the GBS method developed by Elshire et al. (2011). Sample preparation and sequencing were done by the Institut de Biologie Intégrative et des Systèmes (IBIS) at Université Laval (Québec City, Québec, Canada). Two restriction enzymes (PstI/MspI) designed by Poland et al. (2012) were used to digest previously extracted DNA and reduce the complexity of the genome. A GBS library was prepared from 157 individuals from 17 localities (five to thirteen individuals by locality) and sequenced on five Ion Torrent pIv3 chips on an Ion Proton System. All libraries were sequenced by the genomic analysis platform of the Institute of Integrative Biology and Systems (IBIS, Université Laval, Québec, Canada) with a median target of 80 million single‐end reads (50–220 bp) per chip.

### 2.8 Bioinformatics and genetic analyses

For COI, measures of haplotype (*H*) and nucleotide (*π*) diversity were generated using ARLEQUIN v3.1 (Excoffier et al., 2005). Tajima’s *D* (Tajima, 1989) and Fu’s *F*_S_ (Fu, 1997) neutrality tests were performed to determine whether populations presented cases of expansion or a bottleneck of their genetic diversity. The fixation indices (*F*_ST_) were calculated to compare each pair of populations based on haplotype frequency. Networks to depict relationships among haplotypes were also produced using Pop Art with a statistical parsimony network (TCS network) (Clement et al., 2002). To investigate for genetic differences between carrot weevil populations from different regions or host plants, AMOVAs were performed using ARLEQUIN v3.1 (Excoffier et al., 2005). Finally, we tested for correlations between *F*_ST_ and geographic distance using the Mantel test with 1000 permutations in ARLEQUIN.

GBS reads were processed using the software Stacks v.2.54 (Catchen et al., 2013). Raw sequences were demultiplexed and filtered for quality using the process_radtags function (parameters set to ‐r ‐c and ‐q). The reads were then aligned to *L. oregonensis* draft reference genome (GenBank: JAHBCN000000000) using BWA (Li, 2013). Sequence polymorphisms were called and attributed to each population using the function gstacks (‐m), with the parameters set to tolerate, considering all populations combined, a maximum of 10% missing data per locus (‐R 0.9), a minimum minor allele frequency of 5% (‐min‐maf 0.05), and a minimum minor allele count of 3 (‐min‐mac 3). Read coverage was manually checked among samples to avoid low coverage individuals and ensure genotype accuracy (mean per sample coverage = 23×). Genetic parameters were computed using the populations program in Stacks. Genomic diversity was estimated according to the observed heterozygosity (HO), expected heterozygosity (HE), nucleotide diversity (*π*), and the inbreeding coefficients (*F*_IS_). *F*_ST_ values were calculated using the fstats function to compare each pair of populations for each variable site (Meirmans & Hedrick, 2011). Fisher’s exact test was also computed for each *F*_ST_ value. Genetic differentiation between and among populations was visualized using a principal component analysis (PCA) with the function dudi.pca from the ade4 package (Chessel et al., 2004) and a discriminant analysis of principal components (DAPC) with the function dapc from the adegenet package (Jombart, 2008; Jombart et al., 2010) in R (R Core Team, 2020). Admixture was evaluated by calculating the posterior membership probability (probability of each individual to belong to predetermined populations) from the DAPC results. AMOVAs were also performed to explore genetic differences between carrot weevil populations associated with different regions or host plants using the function poppr.amova from the poppr package (Kamvar et al., 2014). Correlation between the genetic (*F*_ST_) and the geographic distance using the Mantel test with 1000 permutations was performed using the adegenet package.

### 2.9 Outlier detection and selection by host plants

Outlier analyses were conducted on the populations from Québec to detect correlations between SNPs putatively under selection and the host plant. First, we used a Bayesian method implemented in the software BayeScan, version 2.1 (Foll & Gaggiotti, 2008), that assumes a Dirichlet distribution of allele frequencies between populations (Foll, 2012). This program estimates the probability that each locus is subject to selection by using a logistic regression on the two locus‐population *F*_ST_ coefficients. We used BayeScan with the default parameters, with a minimum number of iterations set to 50,000, the length of 20 pilot runs to 10,000 iterations, and the burn‐in length to 50,000 iterations. The decision thresholds to call a SNP as being under selection were a q‐value (analog to a false discovery rate *p*‐value) under 0.05 and a posterior probability (comparison with a neutral model) above 0.91, which correspond to a strong relationship on the Jeffreys scale (Foll, 2012). Second, we used a multivariate approach based on discriminant analysis of principal components (DAPC) to identify the specific SNPs most influential in separating samples based on the host plant. The number of clusters in DAPC was set a priori based on the host plant (*k* = 4). Then, a locus‐specific loading plot was created using a threshold of 0.001 to identify the SNPs that most contribute to separating individuals on the first two components. Additional PCAs were produced using only the outlier SNPs. Then, the position of the outlier SNPs was retrieved from the genome assembly and compared for both methods. For the SNPs located in predicted genes, their putative function was determined by comparing the coding sequence with the National Center for Biotechnology Information (NCBI) protein database by means of BLASTX (Altschul et al., 1990) on the nonredundant (nr) sequence database using an *E*‐value significance cutoff of 1e−5.

## 3 RESULTS

### 3.1 Genome assembly and annotation

Whole‐genome sequencing generated just over 35 M nanopore reads, with an average read length of 2123 bp, and an additional 426 M paired‐end Illumina reads. With a combined coverage depth of 165.22×, the assembled genome was 1,293,280,834 bp in length, similar to the size estimated using flow cytometry that ranged between 1356.5 ± 0.4 Mbp in males (*N* = 6) and 1367.4 ± 0.7 Mb in females (*N* = 6). This draft consisted of 41,689 contigs with a L50 of 5172 contigs and an average G + C content of 30.95% (Table S2). Genome completeness, following the analysis of 1013 conserved arthropod genes, was estimated at 82.6% (Table S2).

### 3.2 COI

We obtained 220 sequences of 685 bp of the partial COI from 17 populations of *L. oregonensis* collected from Québec, Ontario, Nova Scotia, and Ohio. Fourteen samples were withdrawn from analyses due to poor DNA quality, and a single individual from one field was not used in our data set. DNA sequence data and specimen collection information were deposited in the Barcode of Life Database (BOLD), under the project ‟Carrot weevil population geneticsˮ (CAWE001‐20–CAWE206‐20). Corresponding GenBank accession numbers are MW471412–MW471617.

**FIGURE 2:**
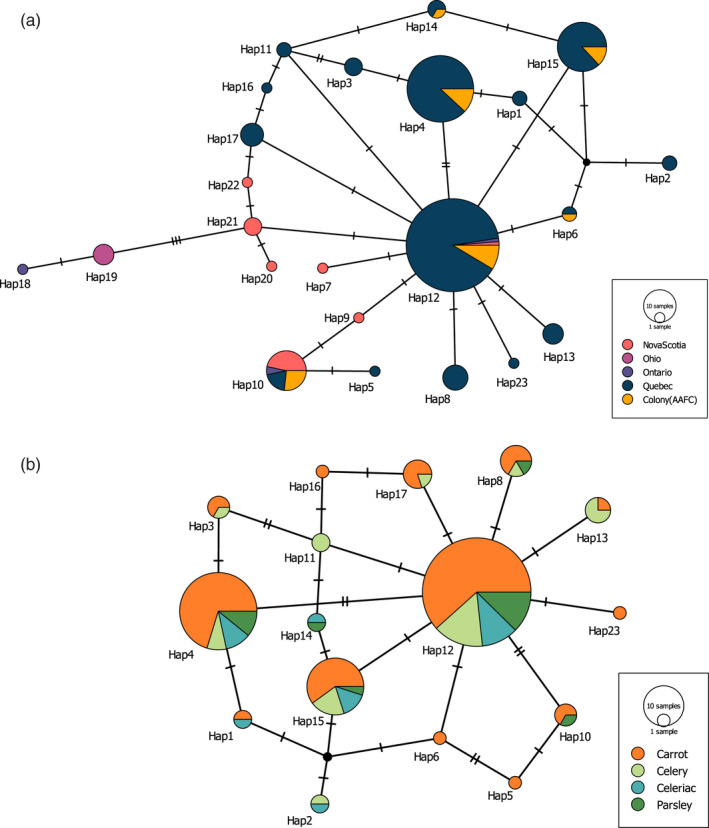
COI haplotype network of *Listronotus oregonensis* from (a) three Canadian provinces and one state in the United States (Ohio) and (b) four host plants from Québec populations. Circle size is proportional to haplotype frequency. Each line segment represents a single mutation between haplotypes

Eighteen polymorphic sites (*S*) were observed, with a haplotype diversity (*H*) ranging between 0.562 and 0.964 (Table 1). The haplotype diversity was high in each population, except for samples collected in Ohio having the same unique haplotype. The nucleotide diversity (*π*) was low for all populations and ranged from 0.002 to 0.003. A total of 23 haplotypes were identified (Hap1–Hap23) across carrot weevil populations (Figure 2a; see Table S3 for detailed haplotype information per site). Hap12 and Hap4 were the most frequent haplotypes shared by 14 and 12 populations, respectively. From all individuals sampled, ten unique haplotypes were identified. Only Hap10 and Hap12 were shared by all geographic regions, except Ohio. Sites in Nova Scotia were those having the most different haplotypes compared with any other populations, with five unique haplotypes. The rearing colony population shared six haplotypes with field populations of Québec.

**TABLE 1:** Sample size (*n*), number of haplotypes (*k*), number of polymorphic sites (PS), haplotype diversity (*H*) ± SD, and nucleotide diversity (*π*) ± SD; results of Tajima’s *D* and Fu’s *F*_S_ neutrality tests with *p*‐values for each *Listronotus oregonensis* population analyzed with COI analysis

Population	*n*	*k*	ps	*h*	*π*	Tajima’s		Fu’s	
						*D*	*p*	*F*_S_	*p*
NE‐AV	6	4	4	0.800 ± 0.172	0.003 ± 0.002	0.15	0.604	−0.69	0.188
NE‐CM	9	5	5	0.750 ± 0.112	0.003 ± 0.002	0.63	0.739	0.51	0.597
OH	4	1	0	—^a^	—	—	—	—	—
ON	3	3	7	—	—	—	—	—	—
QC‐AAFC	21	6	7	0.810 ± 0.048	0.003 ± 0.002	−0.16	0.461	−0.53	0.351
QC‐D	16	4	4	0.692 ± 0.074	0.002 ± 0.001	0.40	0.718	0.44	0.613
QC‐DE	7	3	3	0.714 ± 0.127	0.002 ± 0.001	−1.49	0.051	**−2.31** ^b^	**0.020**
QC‐DP	16	5	5	0.717 ± 0.095	0.002 ± 0.002	−0.30	0.349	0.26	0.494
QC‐GF	19	6	5	0.749 ± 0.086	0.002 ± 0.001	−0.04	0.524	−0.29	0.435
QC‐GP	18	6	6	0.758 ± 0.070	0.002 ± 0.002	−0.24	0.453	−1.52	0.131
QC‐GP2	7	5	5	0.857 ± 0.137	0.002 ± 0.002	−0.49	0.344	−1.29	0.158
QC‐GU	15	5	8	0.562 ± 0.143	0.002 ± 0.001	**−1.74**	**0.031**	−0.88	0.216
QC‐LA	12	5	5	0.788 ± 0.090	0.003 ± 0.002	0.13	0.587	−0.59	0.317
QC‐LE	26	7	8	0.745 ± 0.058	0.002 ± 0.002	−0.76	0.260	−1.42	0.218
QC‐LF	16	6	7	0.717 ± 0.099	0.002 ± 0.002	−0.96	0.178	−1.42	0.135
QC‐LL	8	7	7	0.964 ± 0.077	0.003 ± 0.002	−1.05	0.205	**−4.36**	**0.002**
QC‐VI	2	2	1	—	—	—	—	—	—

^a^ Indices were calculated only on populations with a sample size >5 individuals.

^b^ Values in bold are significant (*p* < 0.05).

Based on the network analysis, Ontario and Ohio were the most genetically distant from Québec and Nova Scotia populations, Nova Scotia being genetically closer to Québec than to other populations (Figure 2a). Populations of Québec showed a greater diversity of haplotypes, certainly resulting from a more exhaustive sampling effort (*N* = 184) compared with other geographic regions (*N* = 20). The AMOVA detected a significant population structure where most of the total genetic variability in carrot weevils was among geographic regions (45.55%) and within populations (sampling locations; 53.60%; Table 3). Neutrality tests (Tajima’s *D* and Fu’s *F*_S_) revealed several negative values (Table 1), suggesting an excess of rare alleles resulting from a recent expansion of these populations, as shown more predominantly by QC‐GU (*D* = −1.74, *p* = 0.031), QC‐DE (*F*_S_ = −2.31, *p* = 0.020), and QC‐LL (*F*_S_ = −4.36, *p* = 0.002).

**FIGURE 3:**
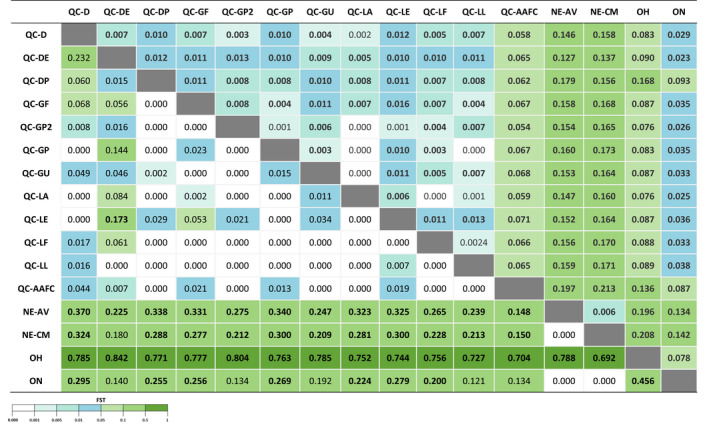
Pairwise *F*_ST_ values among 16 populations of *Listronotus oregonensis* in North America from COI sequences (below diagonal) and GBS analysis (above diagonal). Values in bold are significant (*p* < 0.05)

Pairwise comparisons of *F*_ST_ values in COI were low and nonsignificant in the majority of the Québec samples, indicating low genetic differentiation, except for QC‐DE (*F*_ST_ = [0.000–0.232]) (Figure 3). The population of Ohio showed the highest *F*_ST_ values compared with populations located in Québec (*F*_ST_ = [0.727–0.842]), followed by Nova Scotia (*F*_ST_ = [0.180–0.370]) and Ontario (*F*_ST_ = [0.121–0.295]). The rearing colony population was found to be similar to the field populations in Québec, as also shown by the network analysis (Figure 2a). Isolation‐by‐distance (IBD) tests were significant (*r*² = 0.7682, *p* = 0.01; Figure S1), indicating an increase in genetic differentiation with geographic distance.

No clear haplotype partition was observed among populations of carrot weevils collected on different host plant species in the province of Québec (Figure 2b). Hap4, Hap12, and Hap15 were the most common haplotypes and shared by populations isolated from the four host plant species. Only four unique haplotypes were identified, one on individuals collected from a celery field and the other three from carrot fields. Accordingly, no genetic structure inferred by AMOVA was correlated to host plant species (*p* = 0.751), with most of the genetic variations (98.80%) being observed within sampling locations (Table 4).

### 3.3 GBS

We sequenced 157 individuals from 17 populations of *L. oregonensis* collected from Québec, Ontario, Nova Scotia, and Ohio. Ten samples were removed due to DNA contamination or poor coverage (low number of reads/individual). Paired‐end reads were aligned to the carrot weevil reference genome, which led to the identification of 7393 SNPs. Observed heterozygosity varied little across the populations (Ho = 0.150–0.169), and the lowest value was observed in the rearing colony (QC‐AAFC) with a Ho of 0.150 (Table 2). Nucleotide diversity was high among carrot weevil populations and varied between 0.175 and 0.209, with the lowest values associated with Ohio (0.175) and QC‐AAFC (0.178) populations. The inbreeding coefficient (*F*_IS_) was relatively high and ranged between 0.052 and 0.126.

**TABLE 2:** Sample size (*n*), observed heterozygosity (Ho), expected heterozygosity (He), nucleotide diversity (*π*), and the inbreeding coefficients (*F*_IS_) for each *Listronotus oregonensis* population analyzed with GBS analysis

Population	*n*	Ho	He	*π*	*F*_IS_
NE‐AV	9	0.157 ± 0.002	0.193 ± 0.002	0.205 ± 0.038	0.126 ± 0.009
NE‐CM	9	0.156 ± 0.002	0.191 ± 0.002	0.203 ± 0.038	0.122 ± 0.009
OH	5	0.152 ± 0.003	0.156 ± 0.002	0.175 ± 0.043	0.052 ± 0.006
ON	13	0.159 ± 0.002	0.189 ± 0.002	0.197 ± 0.027	0.126 ± 0.012
QC‐AAFC	8	0.150 ± 0.002	0.166 ± 0.002	0.178 ± 0.034	0.072 ± 0.009
QC‐CLO2	8	0.165 ± 0.002	0.187 ± 0.002	0.200 ± 0.029	0.101 ± 0.009
QC‐D	9	0.162 ± 0.002	0.191 ± 0.002	0.203 ± 0.027	0.124 ± 0.007
QC‐DE	8	0.164 ± 0.002	0.187 ± 0.002	0.201 ± 0.029	0.101 ± 0.009
QC‐DP	9	0.165 ± 0.002	0.182 ± 0.002	0.194 ± 0.028	0.088 ± 0.009
QC‐GF	9	0.155 ± 0.002	0.184 ± 0.002	0.196 ± 0.028	0.120 ± 0.008
QC‐GP	8	0.169 ± 0.002	0.197 ± 0.002	0.209 ± 0.027	0.122 ± 0.006
QC‐GP2	9	0.157 ± 0.002	0.187 ± 0.002	0.200 ± 0.030	0.117 ± 0.008
QC‐GU	8	0.162 ± 0.002	0.184 ± 0.002	0.197 ± 0.030	0.098 ± 0.008
QC‐LA	9	0.159 ± 0.002	0.189 ± 0.002	0.201 ± 0.027	0.125 ± 0.008
QC‐LE	9	0.164 ± 0.002	0.193 ± 0.002	0.205 ± 0.028	0.122 ± 0.007
QC‐LF	8	0.158 ± 0.002	0.184 ± 0.002	0.197 ± 0.030	0.107 ± 0.007
QC‐LL	9	0.154 ± 0.002	0.185 ± 0.002	0.196 ± 0.028	0.122 ± 0.009

**FIGURE 4:**
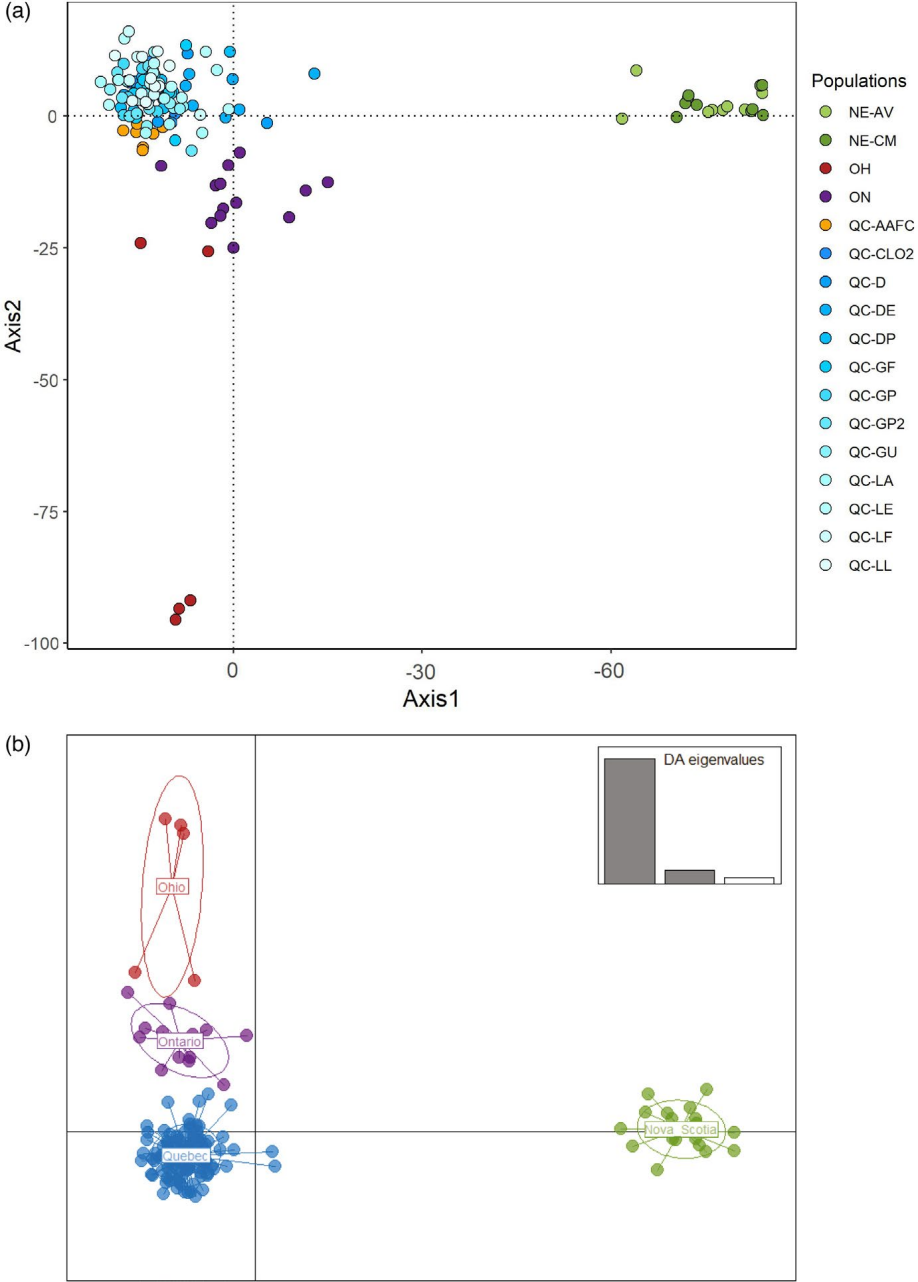
Ordination‐based analyses conducted on SNPs from 17 *Listronotus oregonensis* populations. (a) Principal component analysis (PCA) conducted in all localities: three Canadian provinces (Nova Scotia, Québec, Québec rearing colony, and Ontario) and one state in the United States (Ohio); and (b) discriminant analyses of principal components (DAPC) based on geographic regions. Each point represents one individual, and each color refers to a population (a) or geographic regions (b)

A principal component analysis of the data obtained by GBS revealed a clear partition between the Nova Scotia populations and all the other tested populations (Figure 4a). Although more similar to each other, the populations from Québec, Ontario, and Ohio were clearly clustered by region of origin. Populations from Québec were genetically similar to each other, even when considering the rearing colony (Figure 4a). In addition, a discriminant analysis of the principal components confirmed a strong clustering between geographic regions, reinforcing the pattern of genetic differentiation associated with distance (Figure 4b). This was also supported by the isolation‐by‐distance tests, which showed a good correlation between geographic distance and genetic differentiation (IBD: *r*² = 0.6989, *p* = 0.01; Figure S1). Furthermore, AMOVA indicated a significant contribution of geographic regions in genetic differentiation, but with a smaller percentage of variation (15.92% in GBS compared with 45.55% in COI) (Table 3).

**TABLE 3:** Results of the AMOVA of 17 populations of *Listronotus oregonensis* collected from three Canadian provinces and one US state under the COI and GBS analyses

Source of variation	COI			GBS		
	df	Variation (%)	*p* value^a^	df	Variation (%)	*p* value^a^
Among groups (geographic regions)	3	**45.55**	**0.002**	3	**15.92**	**0.001**
Between samples within groups	13	0.85	0.170	13	**1.94**	**0.001**
Within samples	168	**53.60**	**<0.001**	130	**82.13**	**0.001**

^a^ Values in bold are significant (*p* < 0.05).

**FIGURE 5:**
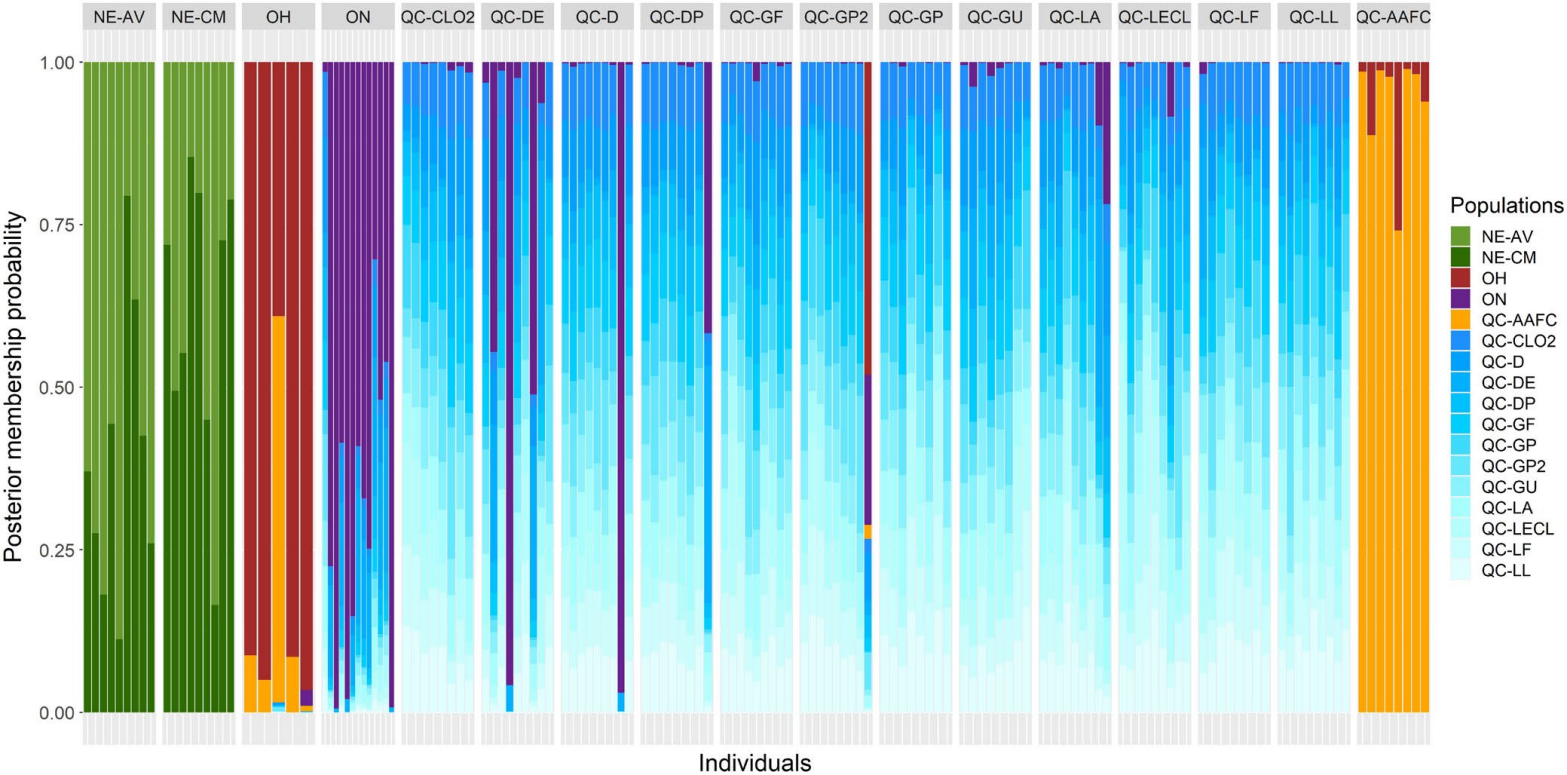
Subdivision of the individuals of *Listronotus oregonensis* based on the DAPC membership probabilities into 17 genetic clusters (one cluster per population)

The membership probabilities of the DAPC analysis for each individual revealed a strong admixture among the populations from Québec and to a lesser degree, with those from Ontario (Figure 5). The two populations from Nova Scotia were admixed together but did not share genotypic information with the other locations. Individuals from Ohio did not show any sign of admixture with other field populations. They shared some information with the rearing colony individuals, suggesting the presence of noninformative loci or heterozygote deficiency due to inbreeding in these populations.

*F*_ST_ values obtained with GBS indicated a low genetic differentiation among Québec populations (*F*_ST_ ranging between 0.000 and 0.013), with the exception of QC‐AAFC (Figure 3). The Ontario population was similar to Québec populations (*F*_ST_ ranging between 0.023 and 0.093), while Nova Scotia (*F*_ST_ ranging between 0.127 and 0.179) and Ohio (*F*_ST_ ranging between 0.076 and 0.168) populations were significantly more differentiated. A linear regression of the *F*_ST_ values obtained with GBS and COI revealed a significant correlation between both genotyping approaches (correlation *R* = 0.78, *p* < 0.001; Figure S2).

**FIGURE 6:**
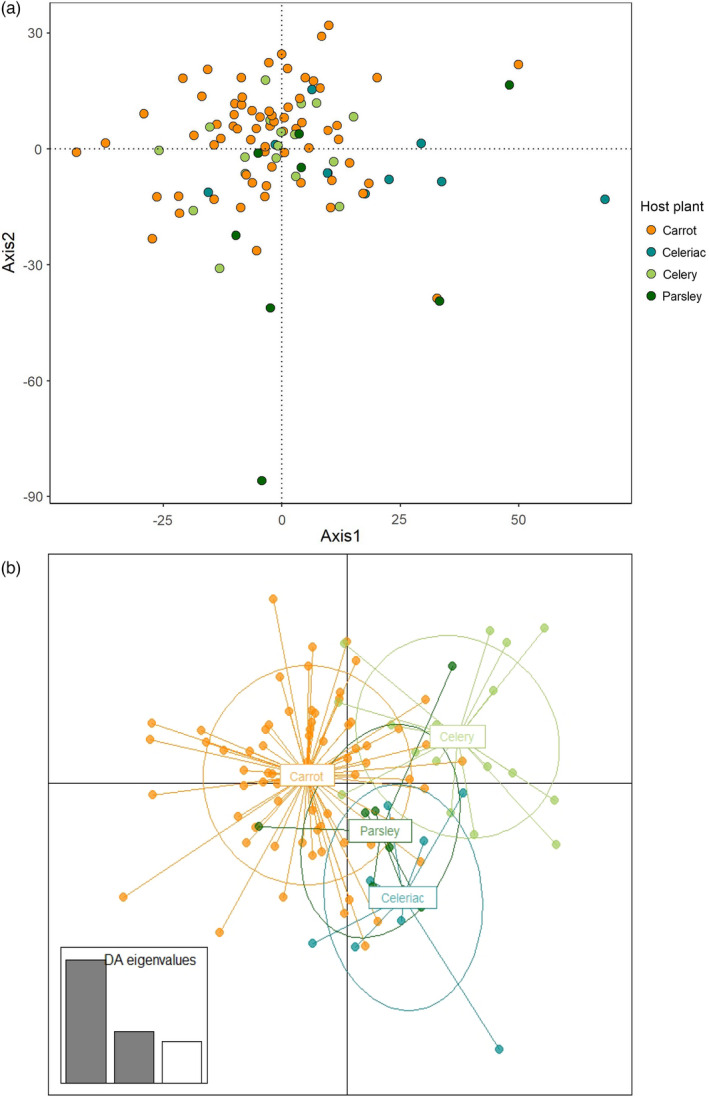
Ordination‐based analyses conducted on SNPs from 12 *Listronotus oregonensis* populations. (a) Principal component analysis (PCA) conducted in the province of Québec with individuals collected on four host plant species (carrot, celery, celeriac, and parsley); and (b) discriminant analyses of principal components (DAPC) based on carrot weevil populations captured on different host plants. Each point represents one individual, and each form (a) or color (b) refers to host plant species

Finally, the comparison of the genotypes obtained by GBS for all individuals from the 12 populations in the province of Québec did not cluster based on their host plants in principal component analyses (Figure 6a). In accordance with COI data, the AMOVA with GBS data revealed no significant impact of host plant species on the genetic differentiation of carrot weevil populations (0.44% variation, *p* = 0.061; Table 4). However, a discriminant analysis using the four host plant species as groups revealed a slight clustering by hosts (Figure 6b) that could indicate an ongoing differentiation process limited to a few genomic regions.

**TABLE 4:** Results of the AMOVA of 12 populations of *Listronotus oregonensis* collected on four different host plants (carrot, celery, celeriac, and parsley) in the province of Québec under the COI and GBS analyses

Source of variation	COI			GBS		
	df	Variation (%)	*p* value^a^	df	Variation (%)^a^	*p* value
Among groups (host plants)	3	−2.30	0.751	3	0.44	0.061
Between samples within groups	8	3.50	0.075	9	**0.66**	**0.011**
Within samples	150	98.80	0.157	90	**98.90**	**0.001**

^a^ Values in bold are significant (*p* < 0.05).

### 3.4 Outlier detection and selection by host plants

The Bayesian method for outlier detection (BayeScan) identified 15 SNPs putatively under selection (Figure S3), while the multivariate analysis (DAPC) highlighted 16 SNPs that contributed the most in clustering the samples based on host plant (7 for the first axis and 9 for the second; Figure S4). The PCAs of these two reduced data sets show a fair clustering of the samples based on their host plant without a clear separation of the four groups (Figures S5 and S6). However, the detailed analysis of allele frequency among each individual for these SNPs did not identify a clear pattern and does not support strong selection (Figures S7 and S8). Interestingly though, one of these SNPs was detected by both methods and located in an intron of a predicted gene. The closest accession (XP_030748181.1) on NCBI was from the rice weevil (*Sitophilus oryzae*) and contained a conserved domain encoding a DDE superfamily endonuclease (pfam03184).

## 4 DISCUSSION

Our study revealed significant genetic differences between distant populations of the carrot weevil across North America, indicating that geographic distance represents a key factor of differentiation. In contrast, no clear evidence of sympatric speciation or genetic differentiation associated with the host plant was observed in the populations of Québec collected from four different cultivated apiaceous plants (carrot, parsley, celery, and celeriac).

Analyses of both mitochondrial and nuclear DNA allowed the characterization of the genetic structure of carrot weevil populations at two timescales. Mitochondrial DNA goes much further back in the evolutionary history of a species compared with nuclear DNA that reveals more recent changes (Avise, 1991; Brumfield et al., 2003). For example, the COI mtDNA network analysis showed that carrot weevil populations from Nova Scotia were slightly more similar to populations from Québec than those from Ontario and Ohio, such a pattern being compatible with a classic stepping‐stone model (Kimura & Weiss, 1964) and supported by IBD results. However, the situation is different in the GBS analysis where the Nova Scotia populations appeared more differentiated from the Québec populations and equally different to other populations. This result suggests a recent genetic differentiation in carrot weevil populations from Nova Scotia and is consistent with their recent detection in carrot fields in this maritime province in 1992 (LeBlanc & Boivin, 1993). This result could also be explained by a lower gene flow between Nova Scotia and other regions due to a geographic barrier where Apiaceous crops are too few to facilitate the movement of the carrot weevil.

Both analyses (mtDNA and GBS) showed great haplotype diversity and complex genetic structure among populations of carrot weevils, which is characteristic of native species (Kim & Sappington, 2004a; Zhang et al., 2018). We also detected heterozygote deficiency in all populations, indicating that populations are locally confined. This pattern could potentially be explained by a limited dispersal capacity of adult carrot weevils and/or the presence of well‐structured populations. The movement of an organism is a fundamental element in ecology and evolutionary processes, which determines its dispersal capacity and distribution area (Nathan, 2008; Nathan et al., 2008). Distribution area is also influenced by biotic and abiotic factors such as resource availability, competition, predation, climate, photoperiod, and landscape (Grez & Villagran, 2000; Renner & Zohner, 2018). The carrot weevil, moving mostly by walking, could also be limited in its dispersal between regions sampled because the apiaceous cultures are not contiguous across the landscape. Like the carrot weevil, the boll weevil (*Anthonomus grandis* Boheman) is not an efficient flyer (McKibben et al., 1991) and hardly flies headway against surface winds (Hardee et al., 1969; Moody et al., 1993). In accordance with our results, Kim and Sappington (2004b) and Raszick et al. (2021) demonstrated using mtDNA and nuclear DNA (ddRADseq) that geographic distance is an important factor of genetic differentiation for boll weevil populations in North America. Following the theory of isolation‐by‐distance, several authors have concluded that differentiation between populations generally increases with geographic distance (Slatkin, 1987). In this study, we found that *L. oregonensis* populations at the regional scale (>1500 km) are isolated by distance, while at the local scale (~50 km), there is a continuous genetic flow among individuals.

At the finer geographic scale, comparison of carrot weevil populations captured from different host plants did not reveal significant genetic differentiation. A slight clustering by hosts was detected by a discriminant analysis on the GBS data, but this result was only partially supported by additional analyses. Two genome scan approaches were compared to identify outlier SNPs linked to host plant differentiation, but only one outlier was detected and shared by the two methods. In addition, the frequency of these alleles varied greatly among individuals collected from a given host and, while the frequency of an allele differed slightly between populations, its presence was not mandatory to develop on a given. Interestingly, the outlier SNP detected by the two approaches was located in a gene with a conserved domain generally associated with transposable elements, which are known to contribute to genome evolution and adaptation in insects (Petersen et al., 2019). Therefore, even if there is no clear evidence of differentiation by host, an ongoing process could be at play. Our results are in accordance with Silva‐Brandão et al. (2015) who showed that host plant associations do not affect the genetic structure of the oriental fruit moth (*Grapholita molesta* Busck), a pest of rosaceous, with geographic distance being a strong driver of population differentiation. For the fall webworm (*Hyphantria cunea* Drury), although geographic distance and host plant were correlated, geographic distance was the main factor contributing to genetic variations between populations (Vidal et al., 2019). However, several mechanisms may promote genetic divergence within sympatric populations that feed on different host plant species (Olivieri et al., 2008). Some insects develop a preference for a given host based on its suitability for development, survival, and reproduction (Hawthorne & Via, 2001; Singer et al., 1988), and others will have different mate choice behaviors depending on the affiliation to a host plant (Feder et al., 1994; Nosil et al., 2007). For example, the milfoil weevil (*Euhrychiopsis lecontei* Dietz) showed a genetic differentiation between individuals that feed on a native host plant, the northern water milfoil (*Myriophyllum sibericum* Komarov), and individuals that prefer a congeneric introduced species, the Eurasian water milfoil (*Myriophyllum spicatum* L.) (Roketenetz et al., 2017). In addition, the apple maggot fly (*Rhagoletis pomonella* Walsh) was previously feeding on the ancestral downy hawthorn (*Crataegus mollis* Torrey and Gray) but developed host shift for the domesticated apple tree (*Malus pumila* Miller), leading to sympatric host race formation (Bush, 1969; Feder et al., 2003). The milfoil weevil and the apple maggot exploit resources that are available throughout the growing season on both wild plants and perennial crops. The carrot weevil, on the contrary, is constrained by annual changes in host plant availability because of crop rotations. This pest species therefore has to exploit border plants, mainly wild Apiaceae species (e.g., wild carrot *D. carota*, wild parsnip *A*. *petroselinum*, wild chervil *Anthriscus sylvestris*), to maintain its populations in the agricultural environment, a pattern that may prevent host plant specialization. However, the impact of wild host plants on the carrot weevil population dynamics remains to be determined. Van Tol et al. (2004) showed that the capacity of the vine weevil (*Otiorhynchus sulcatus* Fabricius) to feed and reproduce on numerous species of less‐preferred host plants contributes to their establishment in different habitats.

To complement the population genetic study, a first carrot weevil genome assembly was generated. The obtained genome indicates a great completeness and quality with a recovering of 82.6% of complete BUSCO genes. The 1.3 GB size for the genome assembly is large compared with other weevil (Coleoptera: Curculionidae) species such as the red palm weevil, *Rhynchophorus ferrugineus* (589 MB) (Dias et al., 2021), and the rice weevil, *Sitophilus oryzae* (770 MB) (Parisot et al., 2021), but similar to a sister species, the Argentine stem weevil, *Listronotus bonariensis* (1.1 GB) (Harrop et al., 2020). As for *L*. *bonariensis*, *L. oregonensis* presents a high proportion of repetitive sequences, explaining the large size of these two genomes. The *L. oregonensis* genome will also facilitate future genetic studies to help management of this pest species.

From an applied perspective, these results help to refine current integrated pest management strategies to control carrot weevil populations. The genotype characterization of the pest populations at the local scale represents an opportunity to find markers of interest that are associated with specific biological functions or biochemical processes. For example, insecticide resistance threatens the success of pest control programs, with carrot weevils from commercial fields in Ontario having developed resistance to the foliar insecticide phosmet (Telfer et al., 2019). Considering that low dispersal capacity of adults constrains gene flow between populations, we might expect a rapid spread of insecticide resistance in carrot weevil populations at local scale following strong selection and limited input of new susceptible individuals. This is all the more worrying given the recent increase in voltinism observed in some areas, which may increase the number of insecticide applications and thus the likelihood of selecting individuals resistant to insecticides. The assessment of insecticide resistance levels in different regions and the identification of associated genetic markers (SNPs) could lead to a tool for rapid molecular detection of resistant populations. Finally, this study opens the way to other avenues of research to identify and understand the factors behind the recurrence and current increase in damage by carrot weevils in apiaceous crops in North America.

## CONFLICT OF INTEREST

The authors have no conflict of interest to disclose.

## Supporting information

Supplementary Material

## Data Availability

Data were archived in different directories: Haplotype data are deposited in BOLD systems under the project ‟Carrot weevil population genetics” (CAWE001‐20–CAWE206‐20) with the corresponding GenBank accession numbers (MW471412–MW471617); the draft reference genome can be found on GenBank (JAHBCN000000000), and GBS reads are deposited in NCBI Sequence Read Archive (SAMN24694696–SAMN24694842).
