# Supplementary material for: Population genetic structure of the carrot weevil (Listronotus oregonensis) in North America
Source: Evol Appl. 2022 Feb 1;15(2):300–15. doi: 10.1111/eva.13343 (PMC8867704; doi:10.1111/eva.13343)
Supplement: Supplementary file 1 — Supplementary Material [file EVA-15-300-s001.docx]

# **Population genetic structure of the carrot weevil (*Listronotus oregonensis*) in North America**

**Supplementary material**

**Supplemental Table 1.** Description of sampling locations, host plants and sampling periods for *Listronotus oregonensis* in North America, and numbers of individuals analyzed using either mitochondrial DNA sequencing (COI) or genotyping-by-sequencing (GBS).

| **Code** | **City, Province/State, Country** | **Spatial coordinates** | | **Geog. regions** | **Host plant** | **Sampling period** | **N of ind. analyzed** | |
| --- | --- | --- | --- | --- | --- | --- | --- | --- |
|  |  | **Latitude** | **Longitude** |  |  |  | **COI** | **GBS** |
| NE-AV | Avonport, Nova Scotia, Canada | 45.1145 | -64.2572 | Nova Scotia | Carrot | June, 2019 | 6 | 9 |
| NE-CM | Cambridge, Nova Scotia, Canada | 45.0517 | -64.6082 | Nova Scotia | Carrot | May-June-July, 2019 | 9 | 9 |
| OH | Homerville, Ohio, USA | 41.0108 | -82.7345 | Ohio | Parsley | March, 2019 | 4 | 5 |
| ON | Guelph, Ontario, Canada | 44.2310 | -79.3551 | Ontario | Carrot | May, 2019 | 3 | 13 |
| QC-AAFC | Saint-Jean-sur-Richelieu, Québec, Canada | 45.1621 | -73.6735 | N/A | N/A | 2018-2019 | 21 | 8 |
| QC-CLO2 | Sainte-Clotilde, Québec, Canada | 45.1621 | -73.6735 | Québec | Carrot | August, 2018 | 0 | 8 |
| QC-D | Sherrington, Québec, Canada | 45.1645 | -73.5792 | Québec | Carrot | May-June, 2018 | 16 | 9 |
| QC-DE | Napierville, Québec, Canada | 45.1811 | -73.3402 | Québec | Carrot | May, 2019 | 7 | 9 |
| QC-DP | Franklin, Québec, Canada | 45.0963 | -73.8765 | Québec | Celeriac | May, 2019 | 16 | 8 |
| QC-GF | Saint-Isidore, Québec, Canada | 45.2436 | -73.6944 | Québec | Celery | May, 2019 | 19 | 9 |
| QC-GP | Sainte-Clotilde, Québec, Canada | 45.1621 | -73.6735 | Québec | Carrot | May, 2019 | 18 | 8 |
| QC-GP2 | Sainte-Clotilde, Québec, Canada | 45.1621 | -73.6735 | Québec | Carrot | August, 2019 | 7 | 9 |
| QC-GU | Sherrington, Québec, Canada | 45.1557 | -73.5905 | Québec | Carrot | May, 2019 | 15 | 8 |
| QC-LA | Sherrington Québec, Canada | 45.1478 | -73.5905 | Québec | Carrot | June-July, 2019 | 12 | 9 |
| QC-LE | Sherrington, Québec, Canada | 45.1387 | -73.5952 | Québec | Carrot | August, 2019 | 26 | 9 |
| QC-LF | Sainte-Clotilde, Québec, Canada | 45.1640 | -73.6699 | Québec | Parsley | May-June-July, 2019 | 16 | 8 |
| QC-LL | Saint-Rémi, Québec, Canada | 45.2512 | -73.6722 | Québec | Celery | May-June, 2019 | 8 | 9 |
| QC-VI | Saint-Rémi, Québec, Canada | 45.2482 | -73.5763 | Québec | Celeriac | May-June, 2019 | 2 | 0 |

**Supplemental Table 2.** Genome assembly and BUSCO statistics for *Listronotus oregonensis* reference sequence (GenBank: JAHBCN000000000)

| # of contig | 41,689 |
| --- | --- |
| Largest contig | 1,457,465 bp |
| Total length | 1,293,280,834 bp |
| GC content | 30.95 % |
| N50 | 61,395 bp |
| N75 | 24,968 bp |
| L50 | 5,172 |
| L75 | 13,479 |
| # N's per 100 kbp | 29.31 |
| BUSCO complete | 82.6 % (81.3 % single; 1.3 % duplicated) |
| BUSCO fragmented | 7.6 % |
| BUSCO missing | 9.8 % |
| BUSCO n | 1,013 |

**Supplemental Table 3.** Haplotype diversity of *Listronotus oregonensis* from three Canadian provinces and one state in USA (Ohio).

| **Name** | **Haplotype**  **28; 37; 109; 130; 132; 178; 182; 205; 268; 301; 322; 331; 337; 387; 394; 478; 631; 635^*^** | **NE-AV** | **NE-CM** | **OH** | **ON** | **QC-AAFC** | **QC-**  **D** | **QC-DE** | **QC-DP** | **QC-GF** | **QC-GP** | **QC-GP2** | **QC-GU** | **QC-LA** | **QC-LE** | **QC-LF** | **QC-LL** | **QC-VI** |
| --- | --- | --- | --- | --- | --- | --- | --- | --- | --- | --- | --- | --- | --- | --- | --- | --- | --- | --- |
| Hap 1 | ACTCATCAATGATGTTTA | 0 | 0 | 0 | 0 | 0 | 0 | 0 | 0 | 0 | 0 | 0 | 0 | 1 | 0 | 0 | 0 | 1 |
| Hap 2 | ACTCATCGGTGATGTTTA | 0 | 0 | 0 | 0 | 0 | 0 | 0 | 1 | 0 | 0 | 0 | 0 | 0 | 0 | 0 | 1 | 0 |
| Hap 3 | ACTCGTCAATGATGTTCA | 0 | 0 | 0 | 0 | 0 | 0 | 0 | 0 | 1 | 0 | 0 | 0 | 1 | 1 | 0 | 0 | 0 |
| Hap 4 | ACTCGTCAATGATGTTTA | 0 | 0 | 0 | 0 | 5 | 6 | 0 | 3 | 2 | 6 | 1 | 2 | 2 | 9 | 4 | 1 | 1 |
| Hap 5 | ACTTATCAGTAGTGTTTA | 0 | 0 | 0 | 0 | 0 | 0 | 0 | 0 | 0 | 0 | 0 | 1 | 0 | 0 | 0 | 0 | 0 |
| Hap 6 | ACTTATCAGTGATGTTTA | 0 | 0 | 0 | 0 | 1 | 0 | 0 | 0 | 0 | 0 | 0 | 0 | 0 | 1 | 0 | 0 | 0 |
| Hap 7 | ACTTGCCAGTGATGTTTA | 0 | 1 | 0 | 0 | 0 | 0 | 0 | 0 | 0 | 0 | 0 | 0 | 0 | 0 | 0 | 0 | 0 |
| Hap 8 | ACTTGTCAGCGATGTTTA | 0 | 0 | 0 | 0 | 0 | 2 | 0 | 0 | 0 | 1 | 1 | 0 | 0 | 0 | 1 | 1 | 0 |
| Hap 9 | ACTTGTCAGTAATGTTTA | 1 | 0 | 0 | 0 | 0 | 0 | 0 | 0 | 0 | 0 | 0 | 0 | 0 | 0 | 0 | 0 | 0 |
| Hap 10 | ACTTGTCAGTAGTGTTTA | 3 | 4 | 0 | 1 | 4 | 0 | 1 | 0 | 0 | 0 | 0 | 0 | 0 | 1 | 1 | 0 | 0 |
| Hap 11 | ACTTGTCAGTGATGTTCA | 0 | 0 | 0 | 0 | 0 | 0 | 0 | 0 | 1 | 0 | 0 | 0 | 0 | 0 | 0 | 1 | 0 |
| Hap 12 | ACTTGTCAGTGATGTTTA | 1 | 0 | 0 | 1 | 7 | 7 | 3 | 8 | 9 | 7 | 3 | 10 | 5 | 10 | 8 | 2 | 0 |
| Hap 13 | ACTTGTCAGTGATGTTTG | 0 | 0 | 0 | 0 | 0 | 0 | 0 | 0 | 3 | 0 | 1 | 0 | 0 | 0 | 0 | 0 | 0 |
| Hap 14 | ACTTGTCGGTGATGTTCA | 0 | 0 | 0 | 0 | 1 | 0 | 0 | 1 | 0 | 0 | 0 | 0 | 0 | 0 | 1 | 0 | 0 |
| Hap 15 | ACTTGTCGGTGATGTTTA | 0 | 0 | 0 | 0 | 3 | 0 | 3 | 3 | 3 | 2 | 1 | 1 | 3 | 2 | 1 | 1 | 0 |
| Hap 16 | ACTTGTTAGTGATGTTCA | 0 | 0 | 0 | 0 | 0 | 0 | 0 | 0 | 0 | 0 | 0 | 1 | 0 | 0 | 0 | 0 | 0 |
| Hap 17 | ACTTGTTAGTGATGTTTA | 0 | 0 | 0 | 0 | 0 | 1 | 0 | 0 | 0 | 1 | 0 | 0 | 0 | 2 | 0 | 1 | 0 |
| Hap 18 | ATCTGTCAGTGACATCTA | 0 | 0 | 0 | 1 | 0 | 0 | 0 | 0 | 0 | 0 | 0 | 0 | 0 | 0 | 0 | 0 | 0 |
| Hap 19 | ATCTGTCAGTGACGTCTA | 0 | 0 | 4 | 0 | 0 | 0 | 0 | 0 | 0 | 0 | 0 | 0 | 0 | 0 | 0 | 0 | 0 |
| Hap 20 | ATTTGTCAGTGATGCTTA | 0 | 1 | 0 | 0 | 0 | 0 | 0 | 0 | 0 | 0 | 0 | 0 | 0 | 0 | 0 | 0 | 0 |
| Hap 21 | ATTTGTCAGTGATGTTTA | 0 | 3 | 0 | 0 | 0 | 0 | 0 | 0 | 0 | 0 | 0 | 0 | 0 | 0 | 0 | 0 | 0 |
| Hap 22 | ATTTGTTAGTGATGTTTA | 1 | 0 | 0 | 0 | 0 | 0 | 0 | 0 | 0 | 0 | 0 | 0 | 0 | 0 | 0 | 0 | 0 |
| Hap 23 | GCTTGTCAGTGATGTTTA | 0 | 0 | 0 | 0 | 0 | 0 | 0 | 0 | 0 | 1 | 0 | 0 | 0 | 0 | 0 | 0 | 0 |
| **Total** | | **6** | **9** | **4** | **3** | **21** | **16** | **7** | **16** | **19** | **18** | **7** | **15** | **12** | **26** | **16** | **8** | **2** |

*Corresponding positions on the sequenced gene

**Supplemental Figure 1.** Isolation by distance (IBD) tests for *Listronotus oregonensis* with A) COI F_ST_ and B) GBS F_ST_. Dgen = Genetic distance between each population (F_ST_); Dgeo= Geographic distance (km) between each population. Populations with low number of individuals (< 3 ind.) were removed from the analysis (QC-VI and ON in COI F_ST_). Regressions were made under a Mantel test based on 999 replicates.

**
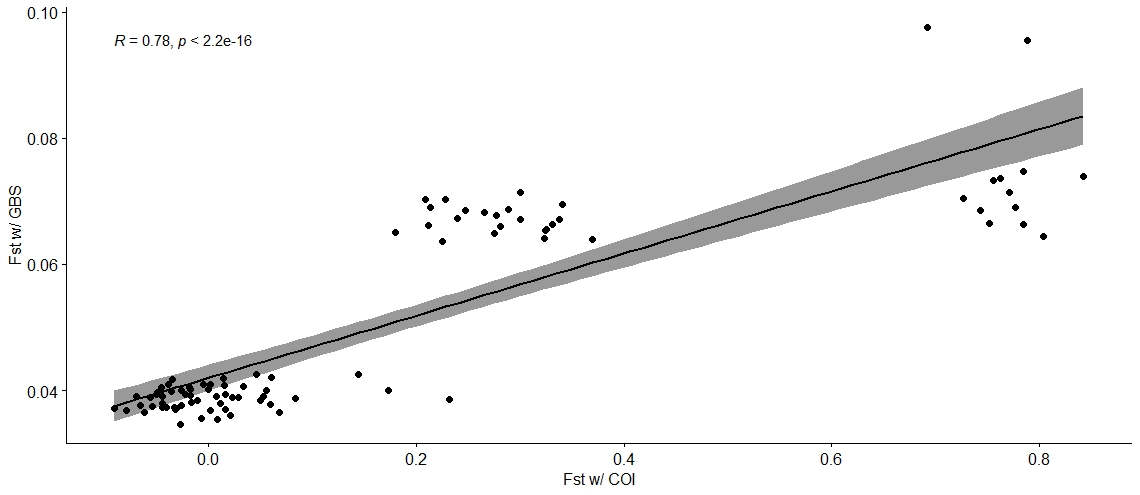
****Supplemental Figure 2.** Correlation between F_ST_ generated with GBS data and F_ST_ from mitochondrial DNA (COI) haplotype analysis with different *Listronotus oregonensis* populations.


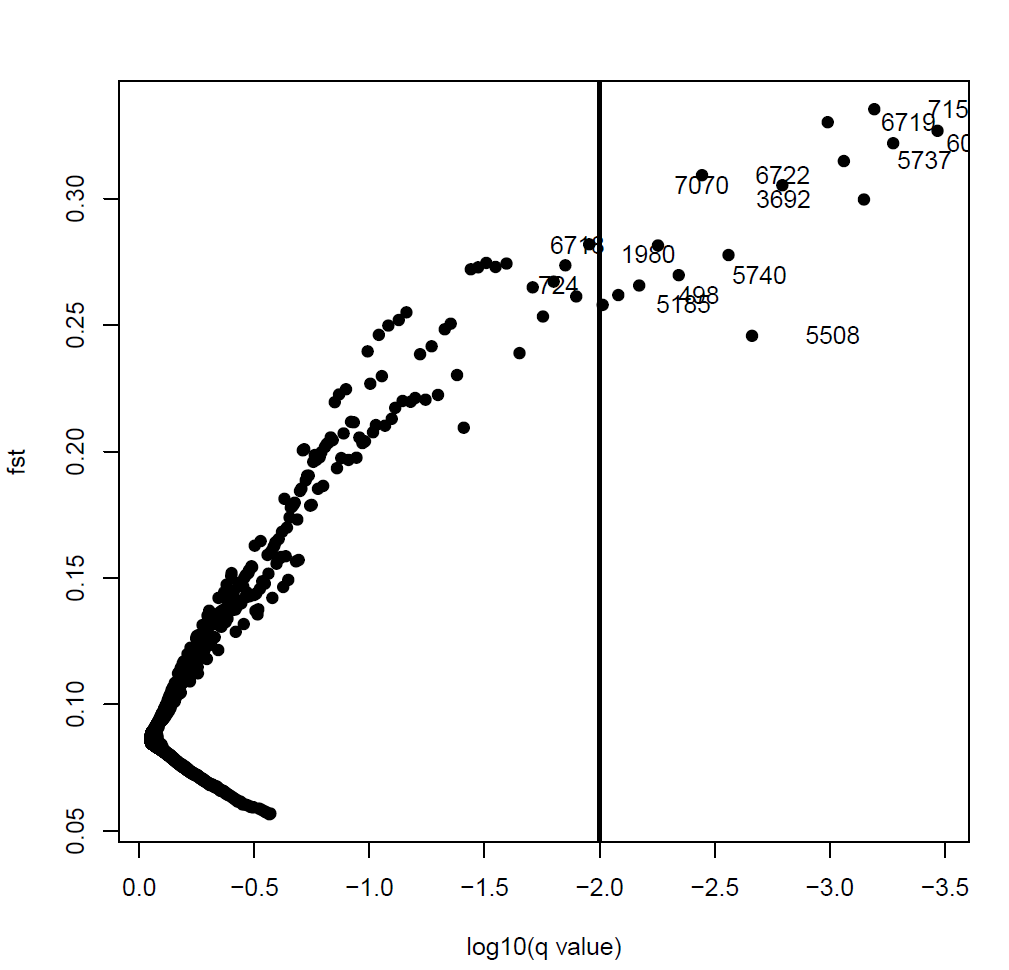


**Supplemental Figure 3.** BayeScan results for the detection of outlier SNP under positive selective (FDR of 5%).


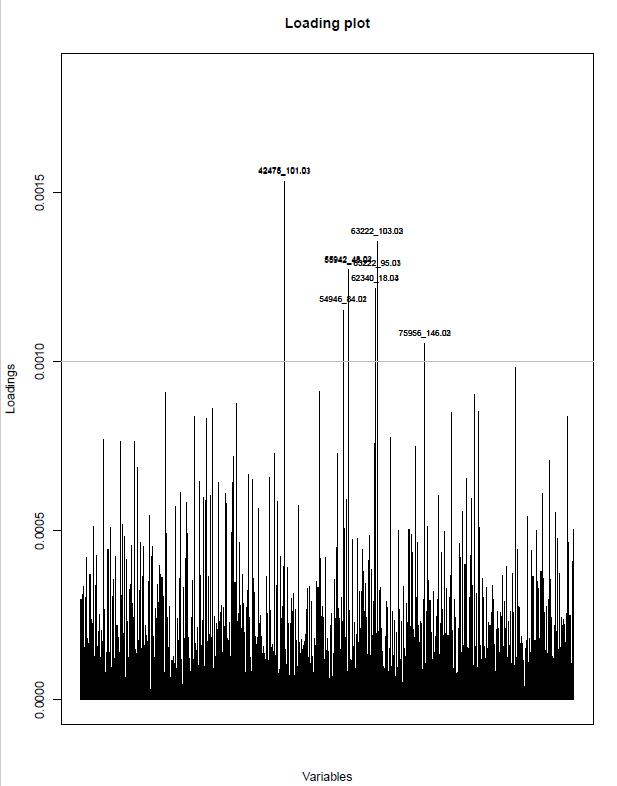

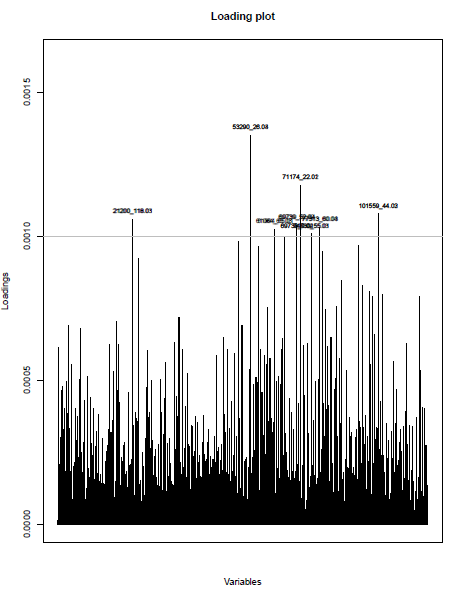


**Supplemental Figure 4.** Loadings of a discriminant analysis of principal components (DAPC) that separate the samples based on their host plant and SNPs that contributed the most to a) axis 1 and b) axis 2.


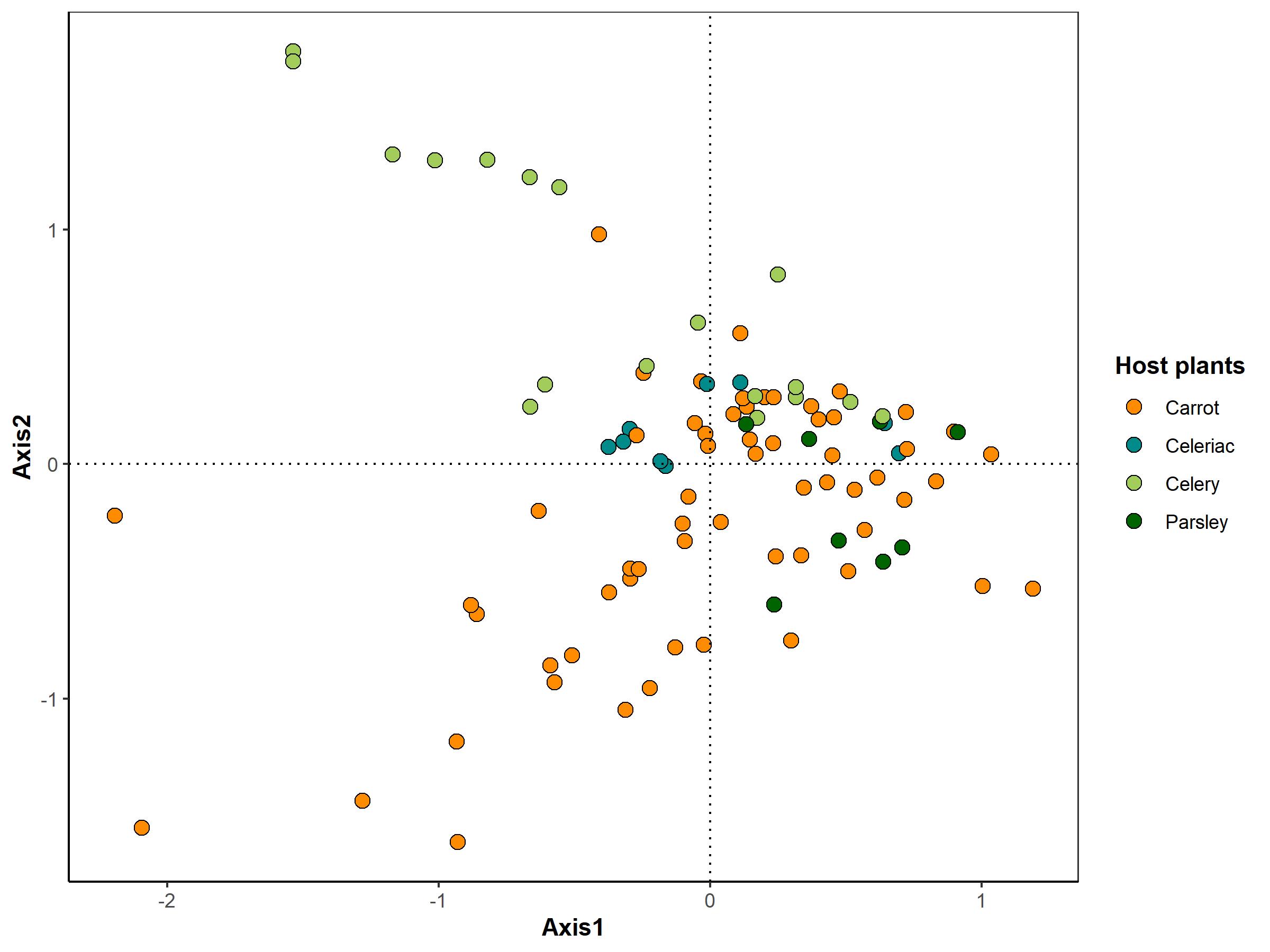


**Supplemental Figure 5.** PCA of the 15 outlier SNPs identified by BayeScan.


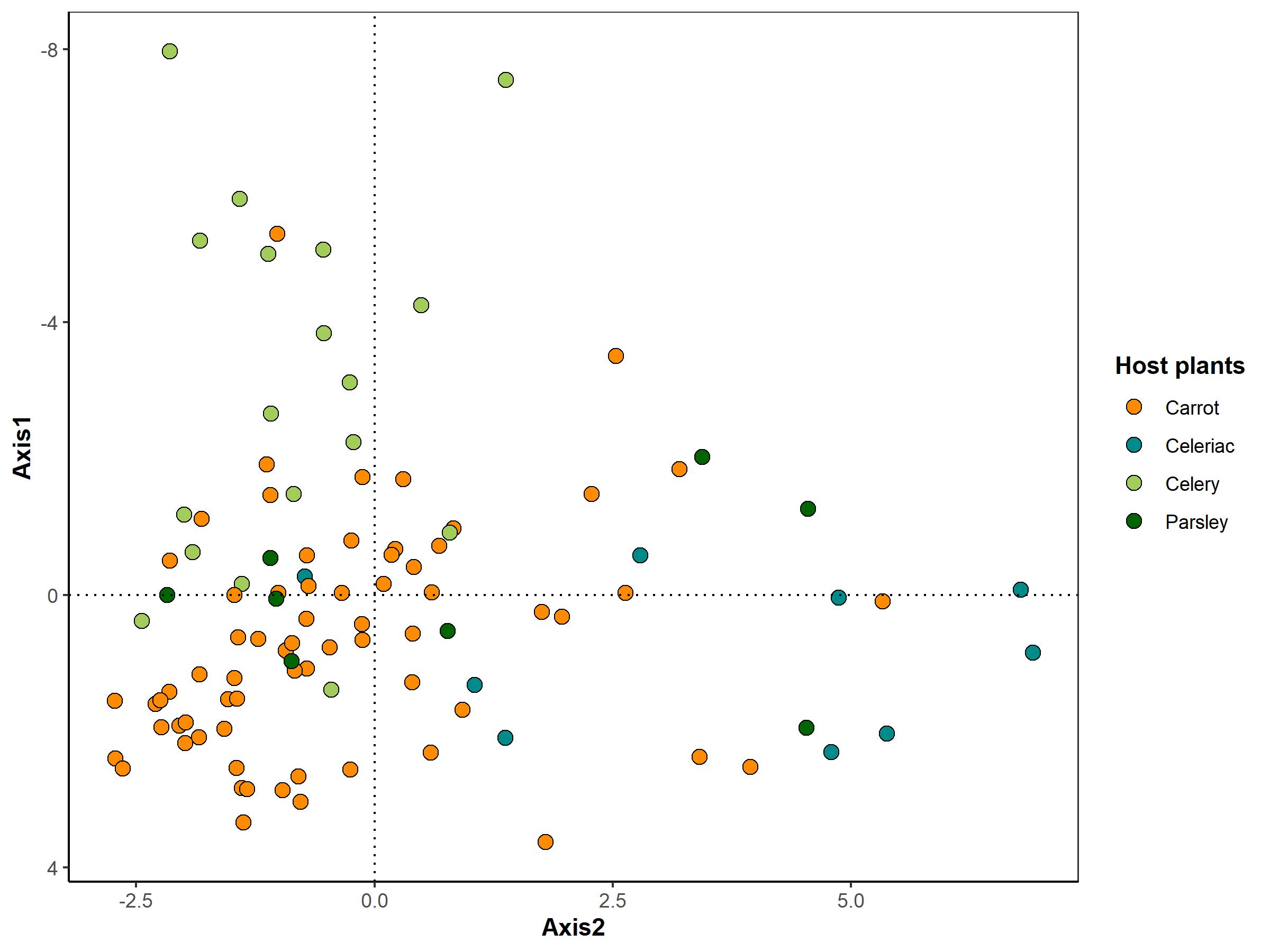


**Supplemental Figure 6.** PCA of the 16 SNPs that contributed the most in clustering the samples based on host plant.


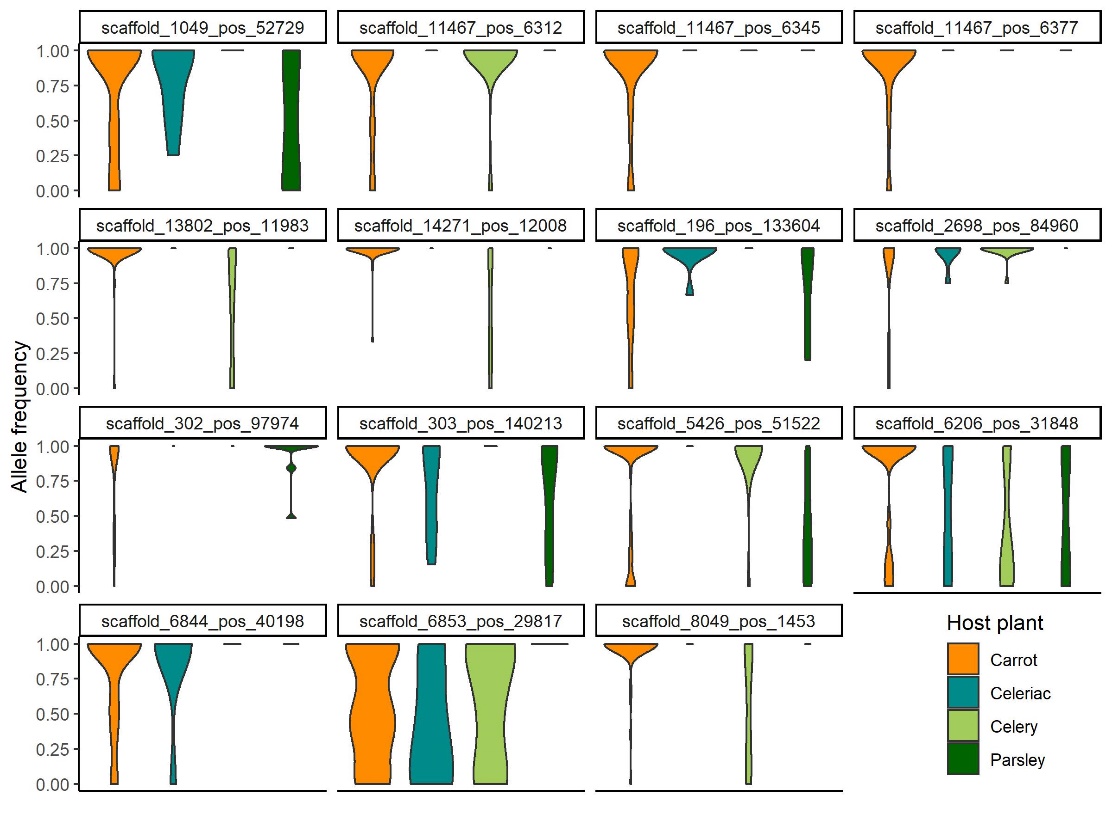


**Supplemental Figure 7.** Allele frequency of the 15 outlier SNPs detected by BayeScan for each sample.


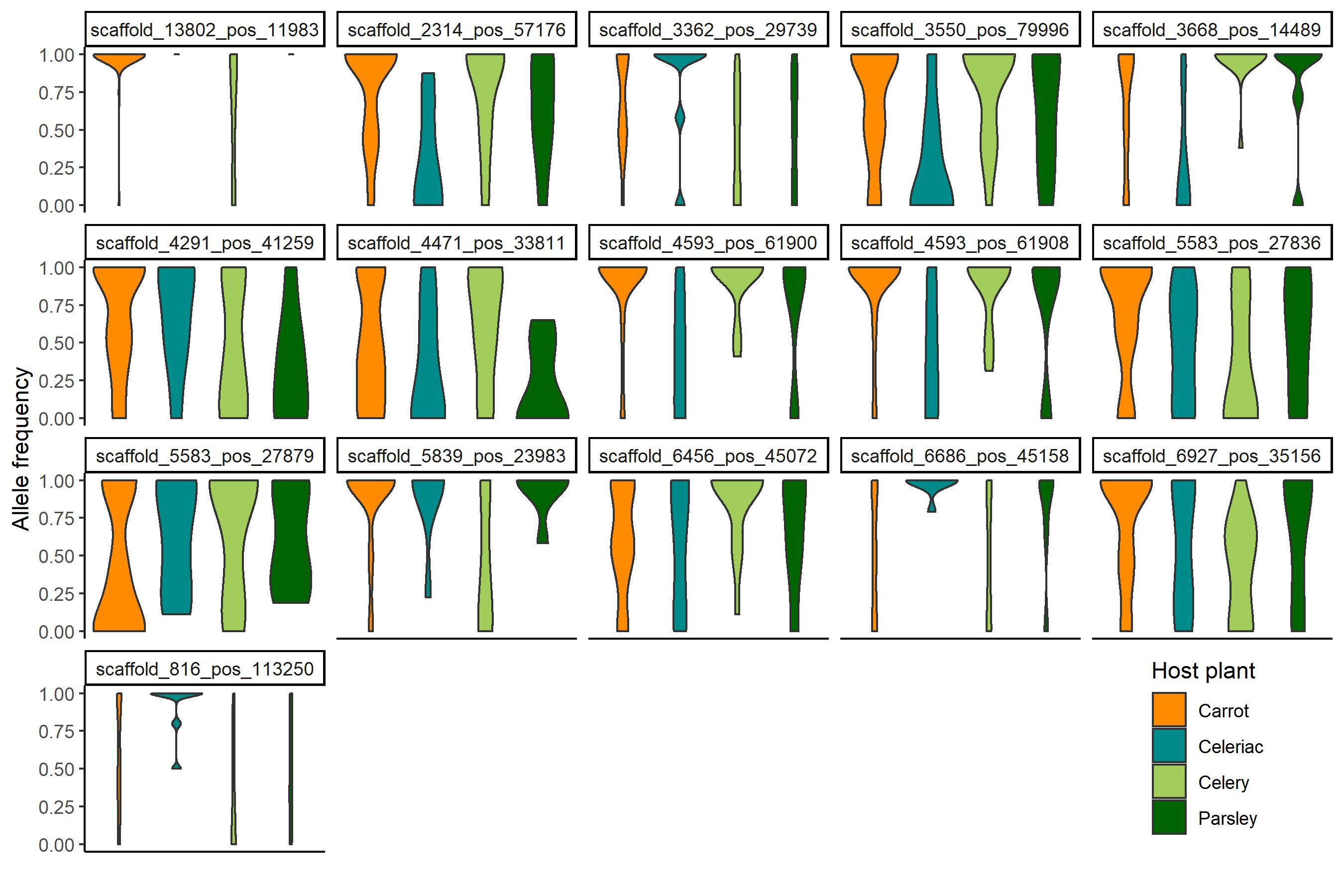


**Supplemental Figure 8.** Allele frequency of the 16 SNPs that contributed the most in clustering the samples based on host plant.
